# Using a microprocessor knee (C-Leg) with appropriate foot transitioned individuals with dysvascular transfemoral amputations to higher performance levels: a longitudinal randomized clinical trial

**DOI:** 10.1186/s12984-021-00879-3

**Published:** 2021-05-25

**Authors:** Chandrasekaran Jayaraman, Chaithanya K. Mummidisetty, Mark V. Albert, Robert Lipschutz, Shenan Hoppe-Ludwig, Gayatri Mathur, Arun Jayaraman

**Affiliations:** 1 Max Näder Lab for Rehabilitation Technologies and Outcomes Research, Shirley Ryan AbilityLab, Chicago, USA; 2 Department of Physical Medicine and Rehabilitation, Northwestern University Feinberg School of Medicine, Chicago, USA; 3 Department of Computer Science and Engineering, University of North Texas, Denton, USA; 4 Department of Biomedical Engineering, University of North Texas, Denton, USA

**Keywords:** Mechanical and microprocessor prosthetic knee, C-Leg, Dysvascular, Transfemoral amputations, Medicare functional classification level

## Abstract

**Background:**

Individuals with transfemoral amputations who are considered to be limited community ambulators are classified as Medicare functional classification (MFCL) level K2. These individuals are usually prescribed a non-microprocessor controlled knee (NMPK) with an appropriate foot for simple walking functions. However, existing research suggests that these individuals can benefit from using a microprocessor controlled knee (MPK) and appropriate foot for their ambulation, but cannot obtain one due to insurance policy restrictions. With a steady increase in older adults with amputations due to vascular conditions, it is critical to evaluate whether advanced prostheses can provide better safety and performance capabilities to maintain and improve quality of life in individuals who are predominantly designated MFCL level K2. To decipher this we conducted a 13 month longitudinal clinical trial to determine the benefits of using a C-Leg and 1M10 foot in individuals at K2 level with transfemoral amputation due to vascular disease. This longitudinal clinical trial incorporated recommendations prescribed by the lower limb prosthesis workgroup to design a study that can add evidence to improve reimbursement policy through clinical outcomes using an MPK in K2 level individuals with transfemoral amputation who were using an NMPK for everyday use.

**Methods:**

Ten individuals (mean age: 63 ± 9 years) with unilateral transfemoral amputation due to vascular conditions designated as MFCL K2 participated in this longitudinal crossover randomized clinical trial. Baseline outcomes were collected with their current prosthesis. Participants were then randomized to one of two groups, either an intervention with the MPK with a standardized 1M10 foot or their predicate NMPK with a standardized 1M10 foot. On completion of the first intervention, participants crossed over to the next group to complete the study. Each intervention lasted for 6 months (3 months of acclimation and 3 months of take-home trial to monitor home use). At the end of each intervention, clinical outcomes and self-reported outcomes were collected to compare with their baseline performance. A generalized linear model ANOVA was used to compare the performance of each intervention with respect to their own baseline.

**Results:**

Statistically significant and clinically meaningful improvements were observed in gait performance, safety, and participant-reported measures when using the MPK C-Leg + 1M10 foot. Most participants were able to achieve higher clinical scores in gait speed, balance, self-reported mobility, and fall safety, while using the MPK + 1M10 combination. The improvement in scores were within range of scores achieved by individuals with K3 functional level as reported in previous studies.

**Conclusions:**

Individuals with transfemoral amputation from dysvascular conditions designated MFCL level K2 benefited from using an MPK + appropriate foot. The inference and evidence from this longitudinal clinical trial will add to the knowledgebase related to reimbursement policy-making.

*Trial registration* This study is registered on clinical trials.gov with the study title “Functional outcomes in dysvascular transfemoral amputees” and the associated ClinicalTrials.gov Identifier: NCT01537211. The trial was retroactively registered on February 7, 2012 after the first participant was enrolled.

**Supplementary Information:**

The online version contains supplementary material available at 10.1186/s12984-021-00879-3.

## Introduction

Estimates show that 54% of all lower limb amputations (LLA) in the USA occur due to peripheral vascular disease [1]. Despite technological advancements in healthcare services and delivery, limb loss due to dysvascular or diabetic conditions is on the rise [1, 2]. With a constant increase in the rate of diabetes leading to peripheral vascular disease, the number of LLA is predicted to increase threefold by the year 2050 [2]. LLA is a life-altering event that negatively impacts individuals’ mobility and ability to be physically active. This diminishes their quality of life, eventually paving the way for psychological issues and other comorbidities [3–6].

A majority of the individuals with transfemoral amputations due to dysvascular conditions are older adults. Furthermore, the combination of vascular disease and aging also results in poor physical strength and balance, impaired neuromuscular coordination, and increased risk for falls. Consequently, it is of prime importance to prescribe an optimal/reliable prosthesis to maximize ambulation safety and restore the ability of older vascular amputees to independently perform physical tasks of daily living [7–10].

A wide spectrum of lower limb prosthetic devices are available to match the ambulation needs of individuals with transfemoral amputation. These include passive mechanical non-microprocessor controlled knees (NMPK) [11], microprocessor-controlled knees (MPK) [12], and more recently powered prostheses [13]. NMPKs offer stability (when aligned properly) for walking on level ground but are less adapted to more dynamically demanding gait scenarios such as walking on uneven surfaces, ramps, and stairs. Such terrains require whole-body biomechanical adaptations to modulate the speed and center of mass to navigate these environments safely. Furthermore, there is also an increased need for dynamic stability and the ability to alter knee stiffness in real-time to assist in both stance and swing phases of the gait, which are not available in NMPKs, leading to higher fall risks [14]. On the contrary, MPKs [15] can alter the knee stiffness depending on the task, to facilitate additional performance during dynamic, real-world situations. Current literature shows that individuals with traumatic transfemoral amputations using an NMPK derive additional performance benefits while ambulating with an MPK [15, 16]. However, research investigating the impact of MPK use on older individuals with transfemoral amputation from dysvascular or diabetic conditions currently using an NMPK is scarce.

In general, the United States Medicare-insured population with transfemoral amputation secondary to vascular disease are not prescribed MPKs. They are traditionally prescribed only to individuals who are considered highly functional (i.e. unlimited community ambulators) and have the potential to ambulate with a variable cadence in most environments (K3–K4 MFCL level) [14, 17]. Most older dysvascular amputees are classified at K1 or K2 level due to their limited ambulation potential and hence are not eligible to acquire an MPK per policy guidance [14, 17]. However, evidence from a few research studies explicitly focusing on this population suggests that older individuals with transfemoral amputations from dysvascular conditions, designated as limited community ambulators (K2 level), may benefit from using an MPK [18, 19].

Despite such research evidence, policy agencies indicate that the quality of these research studies is not adequate. Specifically, the study design limitations are roadblocks for guiding policy level implementations [20]. Some recommendations by policy agencies to improve the quality of study design to facilitate policy level evidence translation were [20], (i) include outcomes specific to dysvascular or diabetic transfemoral medicare population for generalizability, (ii) administer similar co-intervention to all study groups (i.e. providing similar intervention/therapy to both the MPK and the comparative device), (iii) standardize acclimation periods, length of study, and assessment period for activities between MPK and comparative devices, (iv) prospectively set benchmarks for performance outcomes that would determine the success of the research, (v) use research techniques and methodologies that strengthen the overall quality of the study, and (vi) include outcomes that encompass a wide set of clinical performance and self-reported measures.

Therefore to directly address these research gaps, this prospective longitudinal clinical trial was designed to investigate the benefits of using an MPK (C-Leg) and appropriate foot ankle complex in a group of individuals with transfemoral amputation due to dysvascular or diabetic conditions. Based on the policy level recommendations, the study design factors including length of study, acclimation, training, assessment timelines, and co-interventions were standardized.

We hypothesized that providing an MPK C-Leg + appropriate foot will significantly improve their performance in terms of walking/gait speed, balance, and safety with sufficient acclimation for individuals with transfemoral amputees at MFCL K2 level due to vascular disease currently using an NMPK leg. To facilitate subjective validation of this expected outcome, previously published metrics (averages) from the current literature (gait performance, balance, and fall risk safety in participants with transfemoral amputation) were used to compare the improvements in scores to a higher performance level.

## Methods

### Inclusion criteria

Inclusion criteria were: (i) Dysvascular or diabetic unilateral transfemoral amputation, (ii) at least 6 months or more post-prosthetic fitting, (iii) currently using an NMPK + appropriate foot, (iv) household or limited community ambulators post-amputation (MFCL K1 or K2 level).

### Exclusion criteria

Exclusion criteria were: (i) Subjects with amputation secondary to trauma or cancer, or congenital causes were excluded from this study. (ii) Skin lesions/ulcers on the residual limb that could prevent them from physical activity or fitting the prosthesis. (iii) Cognitive deficits or visual impairments that would impair their ability to give informed consent or to follow simple instructions during the study.

### Participants

The study protocol was approved by the local Institutional Review Board (IRB: STU00042823). Study participants were recruited from amputee clinics of the Shirley Ryan AbilityLab (formerly known as Rehabilitation Institute of Chicago) and Northwestern University Orthotics and Prosthetic clinics of the Chicagoland area. Recruitment was based on a convenience sample. A total of 10 participants agreed to participate in the study. All subjects provided voluntary informed consent prior to participation in any study activities. The participants’ medical history was collected.

### Outcome metrics

The outcome metrics for assessment were chosen based on prosthetic literature as comprehensive assessments for performance-based clinical outcomes [21–24] as well as participant-reported outcomes [25].

#### Clinical performance-based measures

Clinical performance-based measures consisted of the 10-m walk test (10MWT), 6-min walk test (6MWT), BERG balance test (BERG), Four Square Step Test (FSST), and the Timed Up and Go test (TUG). These measures were used to assess the participant’s gait and balance capabilities. Amputee Mobility Predictor (AMPPro) was used to assess the mobility of individuals with lower limb amputation and predict functional capability.

#### Participant-reported outcome measures

Participant-reported outcome measures included the following: Modified Falls Efficacy Scale (MFES) and Prosthesis Evaluation Questionnaire (PEQ). They were scored as per the standard scoring guides associated with each tool. These outcome measures assess the user’s confidence level and the quality of life associated with prosthesis use.

## Devices

### Microprocessor knee (MPK)

C-Leg 3C98-3 designed by Ottobock HealthCare^®^ is a microprocessor controlled knee with an integrated sensor system that controls a hydraulic unit’s damping characteristics in real-time to assist in both stance and swing phases of the gait. Integrated sensor data is gathered and evaluated at 100 Hz, allowing for dynamic control of the knee joint (see Appendix).

### Foot

Ottobock’s 1M10 Adjust foot is a multiaxial foot which offers dampening characteristics and allows easy rollover during walking. The flexible, functional module along with the forefoot ball-pad provides stability to the user while standing and walking. The multi-axial nature of the foot allows for inversion/eversion in the frontal plane and plantarflexion/dorsiflexion in the sagittal plane. 1M10 is commercially available and recommended for individuals with amputation at a K2 level of activity.

### Predicate prosthetic knee and foot combination (NMPK)

All subjects used their clinically prescribed NMPK and appropriate foot for the study. The NMPK and foot were all fitted and aligned by a certified and licensed prosthetist. The details are provided in Table 1.

**Table 1:** Participant demographics

SubID	Sex	Age (years)	Height (cm)	Weight (kg)	Etiology	Time since Amp. (years)	Predicate NMPK	Predicate foot	Assistive device	Rand. Ord
P1	F	76	165	72	Circulation problems	3	OWW GeoFlex	Ossur K2 sensation	Rolling walker	0
P2	M	57	173	61	Circulation problems	4	Ossur Mauch SNS	Ossur flex foot assure	None	1
P3	F	54	157	92	DM/circulation problems	1	Ossur total knee	Ossur variflex LP	Bilateral St. Cane/rolling walker	0
P4	F	52	175	70	Circulation problems	28	Ottobock 3R49 (Wt. activated Stance Control)	Trulife seattle lightfoot	None	1
P5	M	61	175	63	HT/circulation problems	2	Ossur Total Knee 2000	OWW SACH	Quad cane	1
P6	F	69	160	63	DM/HT/CAD	3	–	–	Rolling walker	1
P7	M	69	170	84	DM/HT/CAD	8	Hydraulic	–	Rolling walker	0
P8	F	72	152	59	Circulation problems	5	WASC	CPI accent	Rolling walker	0
P9	F	54	163	77	Circulation problems	1	Ossur total knee 1900	Freedom WalkTek	Rolling walker	1
P10	M	70	175	66	Circulation problems	3	Ossur total knee	Ottobock	Rolling walker/crutches	1

DM = diabetes mellitus; HT = hypertension; CAD = coronary artery disease; Rand. Ord = 0–> CLeg then MK (Block 1); Rnd. Ord = 1–> MK then CLeg (Block 2); – = Information unavailable/Not provided

### Clinical trial design

**Fig 1:**
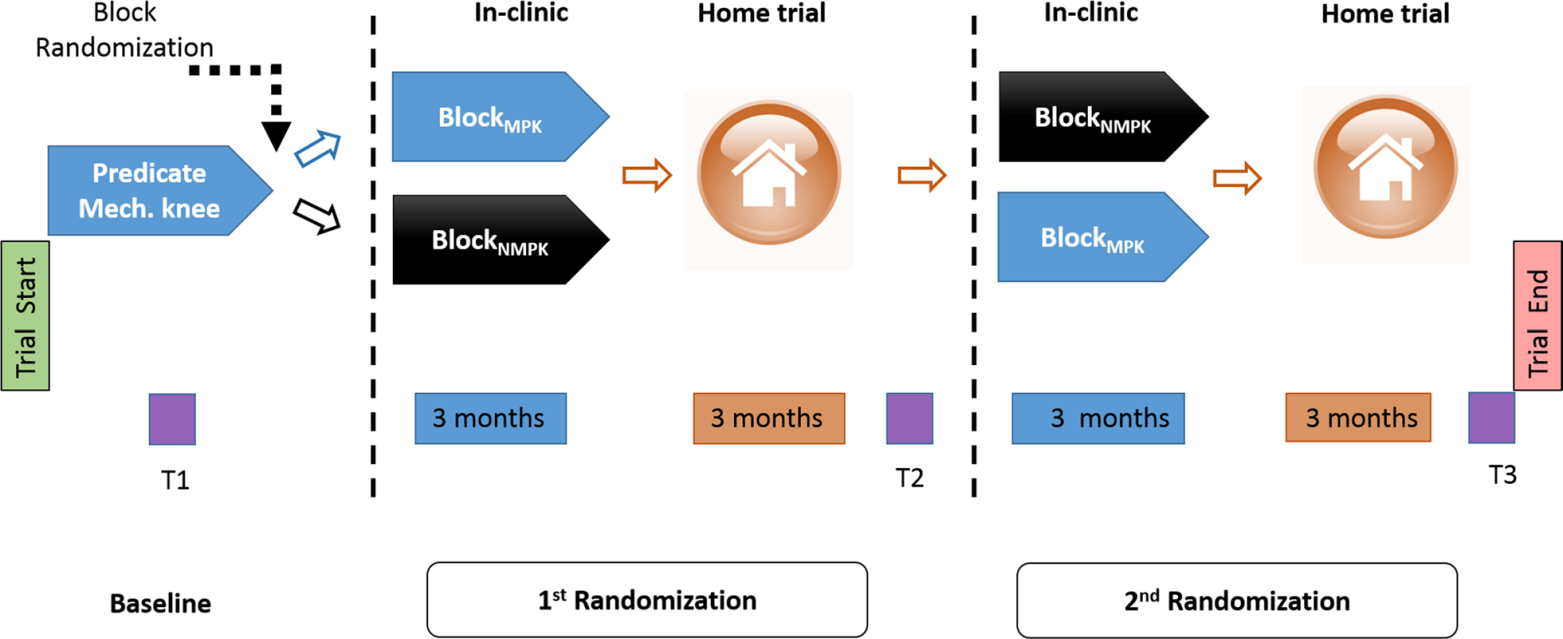
Clinical trial design schematic and outcome assessment time points (T1, T2, and T3)

This prospective longitudinal study was a randomized control clinical trial with a crossover design. This clinical trial was designed to compare the performance of the MPK (C-Leg) + appropriate foot with the participant’s baseline performance (with predicate NMPK leg + predicate foot at baseline). The clinical trial design is shown in Fig. 1.

The sample size of the study was estimated using the primary outcome of the clinical trial, which was a global position system (GPS)-based-metric. The details of the sample size and power estimation can be seen in the “Sample size estimation” section. However, the main focus of this manuscript was to discuss the effect of the prosthetic interventions on clinical performance and participant-reported metrics.

#### Assessment time points

There were three assessment points, namely, assessments at baseline after consent (see Fig. 1, T1), at the end of the randomized Block 1 (see Fig. 1, T2), and at the end of randomized Block 2 (see Fig. 1, T3).

#### Adverse events

Falls and skin-related issues while using the prosthesis were identified as potential adverse events for this clinical trial.

#### Minimizing bias and co-intervention effect

For consistency and to minimize the co-intervention effect, a 1M10 foot type was used for all participants during the intervention duration; the foot size and type were matched to their respective predicate foot for each subject. Participants were asked not to participate in any other research studies or rehabilitation interventions during the study period. To reduce any protocol-related bias to outcome assessments, we standardized the fitting, training protocol for device acclimation, and home trial periods between the interventions (i.e. BlockMPK and BlockNMPK). Further, as the focus is to test the efficacy of the MPK + appropriate foot combination, to minimize any effect of variability due to different foot types, all participants were fitted with the same foot (1M10) for T2 and T3 assessment time points (Fig. 1).

### Intervention randomization protocol

A researcher blinded to the study protocol generated the pseudorandom sequence using MATLAB (Table 1). The random assignments were in a sealed envelope. The personnel who generated the random sequence was not involved in enrolling participants, interventions, or data collection.

#### Block randomization (Fig. 1)

Participants were randomly assigned to one of the two interventions (MPK or NMPK) of this clinical trial. The two intervention were, (i) Block_MPK_: Training for acclimation and testing with the MPK (C-Leg) attached with an appropriate 1M10 foot and (ii) Block_NMPK_: Training for acclimation and testing on their NMPK (current home use device) attached with an appropriate 1M10 foot (see Fig. 1). Group 1 performed the Block_MPK_ followed by Block_NMPK_ schema and group 2 followed the reversed schema (i.e. Block_NMPK_ followed by Block_MPK_). The total study duration was 13 months. There was no washout period between assessment time points (T1, T2, and T3).

#### Baseline

Prior to randomization into the clinical trial, participants were assessed with a full battery of baseline outcome measures (see “Outcome metrics” section). For baseline assessment all participants used their current home-use prosthetic knee and appropriate foot (predicate device).

Post-baseline evaluation, participants proceeded for fitting and device training for acclimation based on their randomized block assignment (Block_MPK_ or Block_NMPK_). If the participant was first assigned to Block_MPK_, both their NMPK knee and foot type were switched respectively (i.e. predicate NMPK switched to a C-Leg and predicate foot to 1M10 foot). Alternatively, if the participant’s first random assignment was to Block_NMPK_ only the predicate foot was switched to 1M10 and their predicate NMPK was left unchanged.

#### Use of assistive devices

Participants were allowed to use their assistive devices such as a cane, walkers, crutch, etc., during the acclimation sessions as well as during the outcomes testing. Our protocol did not restrict the use of assistive devices during the entire duration of study participation. We allowed assistive devices to be used as needed by the participants. The details of assistive devices used can be found in Table 1.

#### First randomized Block

##### Prosthetic device fitting and training for acclimation (3 months)

All fitting and adjustments were carried out at the Rehabilitation Institute of Chicago (currently known as Shirley Ryan AbilityLab). A certified and licensed prosthetist performed all fitting and socket adjustments. A prosthetist assured that the fitting was appropriate and acceptable to the participant’s comfort level (verbal feedback and approval) in both intervention cases. Post-fitting, each participant went through a structured training protocol to acclimate to their changed devices. The structured acclimation regime included training for device operation and safety, strength, balance, endurance, walking on different surfaces (uneven, ramps, stairs), and tasks related to other activities of daily living. Individualized sessions were carried out by a licensed physical therapist. Each participant trained for 1–3 sessions a week (each session ~ 1–2 h) during this phase for a maximum of 14 sessions over three months. The training regime was designed to provide adequate adaptation time for the individuals to use the device independently and safely in a home and community environment.

##### Home trial (3 months)

After acclimation, participants left with their assigned device, either Block_MPK_ or Block_NMPK_. For the home trial, participants were asked to use the intervention device for their day-to-day community mobility and while performing activities of daily living during the three months.

##### Post home trial assessment (Point T2, Fig. 1)

At the end of the first home trial period, participants returned to the clinic and were assessed (T2) with the same battery of outcomes as in T1 assessments ("Outcome metrics" section above).

#### Second randomized Block

After completion of their first randomized Block, participants switched to their second Block (i.e. perform Block_NMPK_ if started with their first randomization intervention with Block_MPK_ or vice versa). Participants followed the same protocol for fitting, acclimation (3 months), home trial (3 months) period, and post home trial assessments as performed in their first randomized block to complete their participation in the clinical trial.

### Data analysis

#### Blinding of assessment/data analysis

At the completion of both interventions, all outcomes measures collected during the clinical trial were aggregated and analyzed by personnel blinded to the clinical trial hypothesis and intervention procedures.

#### A comparison of study clinical outcomes to previous literature

To compare clinical performance improvements obtained from the current prosthetic interventions (i.e. a potential improvement in performance metrics matching higher MFCL level such as K3) the following metrics (means) from previously published literature in individuals with transfemoral amputation were used [17]. Gait speed: MFCL K3 = 0.88 ± 0.39 m/s [24]. Distance covered during the 6MWT: MFCL K3 = 311.30 ± 112.29 m [24]. Berg Balance Scale (BBS): K2 ≤ 50/56 [26]. PEQ-MS: K2 = 63 and K3 = 79 [27]. MFES: 9.8 for healthy [28]. AMPPro: MFCL K3 = 40.5 ± 3.9 [29]. In addition, when available, transfemoral amputation population-specific minimum detectable change (MDC_90%_) from literature was also used to make objective clinical inference on improvements between baseline and the interventions (AMPPro) [29, 30].

#### Clinical trial design statistical quality assessment

To assess the quality of the clinical trial design the ‘Intervention Statistical Validity Rating of Methodological Quality’ according to Hofstad et al. [31] commonly employed in prosthetic literature was used [18]. The procedure of scorings and related details are provided in “Clinical trial quality assessment” section.

#### Statistical analysis of clinical trial outcomes hypothesis-test design

SigmaPlot 14 (Systat Software Inc., UK) was used for all statistical analyses. A series of Shapiro–Wilk and Brown–Forsythe tests were conducted to assess if the data were normally distributed and had equal variance respectively. All variables passed the Shapiro–Wilk normality test (P’s > 0.05). Following this, a series of generalized linear model (GLM) two-way repeated measures ANOVA’s were performed on each of the outcome variables to test our hypotheses. The intervention (device) type and randomized experimental order were used as the fixed factors in the ANOVA. Bonferroni correction was applied to account for multiple comparisons. If the GLM test showed statistical significance, it was followed by a posthoc pairwise t-tests to ascertain if there were any statistical group effects between baseline vs MPK C-Leg + 1M10 foot and baseline vs NMPK + 1M10 foot. Per design, there was no direct statistical comparison between NMPK and MPK C-Leg outcomes. The P-value was set to P ≤ 0.05. Since the predicate NMPK types were varied between participants, each participant was treated as his/her control in this pairwise comparative design.

## Results

From here on for brevity, ‘baseline’ implies predicate NMPK with predicate foot type, ‘MPK C-Leg’ implies group averaged result for MPK C-Leg fitted with 1M10 foot and ‘NMPK’ implies group averaged result for predicate NMPK fitted with 1M10 foot.

### Participants

The detailed participant demographics (n = 10: 4 Male/6 Female; mean age = 63 ± 9 years; mean height = 166.6 ± 8.3 cm; mean weight = 71 ± 11 kg; 50% (n = 5) of the group were older adults aged 69 and above. Participants had a mean time of 5.8 ± 8.1 years since their amputation and further demographic information is provided in Table 1. No adverse events were reported during the study duration. All participants acknowledged that they did not participate in any other research study or intervention during their active study period (13 months).

### Clinical performance-based measures

Statistically significant improvements were seen for the 10MWT, a measure of gait speed (in seconds): [F(2,26) = 3.92, P = 0.045, power = 0.48, baseline = 22.3 (9.7) s, MPK C-Leg = 15.4 (7.3) s, NMPK = 18.0 (7.8) s]_10MWT time_, [F(2,26) = 6.77, P = 0.009, power = 0.78, baseline = 0.48 (0.15) m/s, MPK C-Leg = 0.76 (0.28) m/s, NMPK = 0.66 (0.29) m/s]_10MWT speed_. Follow-up posthoc pairwise tests revealed that the participants significantly improved in their 10MWT performance when using the MPK C-Leg in comparison to the baseline [t(16) = 2.77, P = 0.046, baseline = 22.27 (9.72) s, MPK C-Leg = 15.4 (7.3) s, NMPK = 18.0 (7.8) s]_10MWT time_; [t(16) = 3.65, P = 0.008, baseline = 0.48 (0.15) m/s, MPK C-Leg = 0.76 (0.28) m/s, NMPK = 0.66 (0.29) m/s]_10MWT speed_. Participants showed no statistically significant improvement from the baseline condition for the 10MWT performance when using their NMPK (P’s > 0.05). These results signify that MPK C-Leg combination provided enhanced performance benefits in terms of improved gait performance compared to the NMPK.

No statistically significant differences were observed for the 6MWT [group averages in meters: baseline = 137.5 (85.6), MPK C-Leg = 145.2 (110.3), NMPK = 147.5 (112.0)], BERG [baseline = 37 (8), MPK C-Leg = 44 (13), NMPK = 39 (15)], TUG in seconds [baseline = 27.5 (15), MPK C-Leg = 25.3 (14.1), NMPK = 29.0 (16.3)], and FSST in seconds [baseline = 17.4 (5.0), MPK C-Leg = 16.8 (11.2), NMPK = 19.6 (12.4)]. There were no significant interactions between the device type and the randomized order (GLM F-tests all P > 0.05) for these clinical assessments.

### Clinical relevance

#### Gait speed

**Fig. 2:**
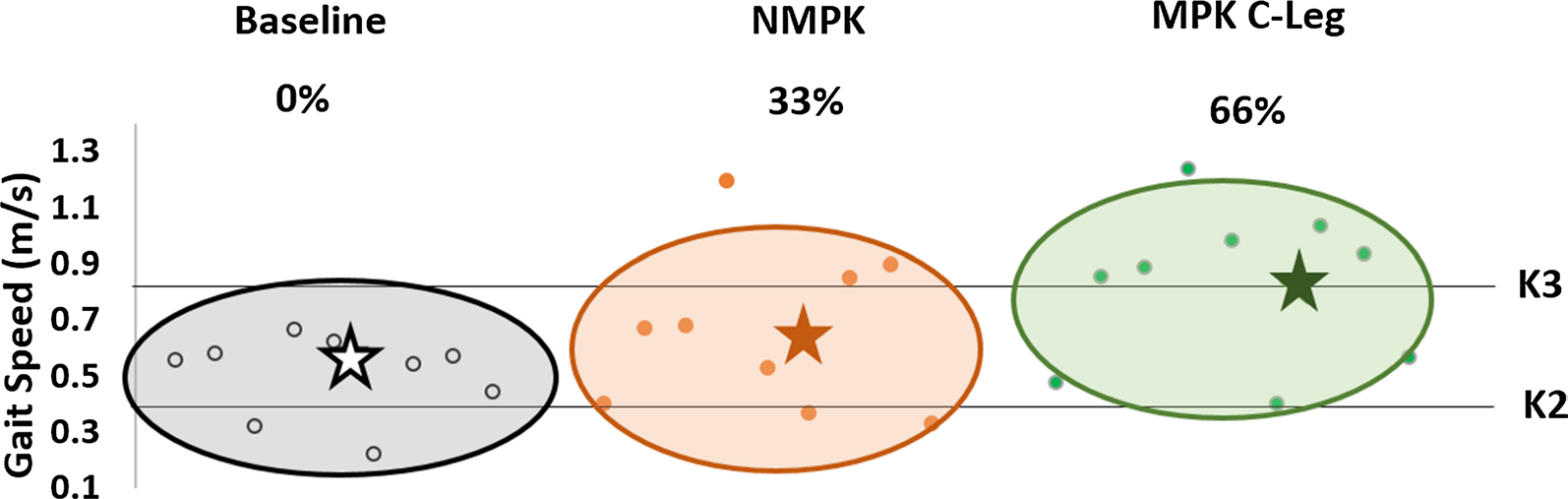
Performance improvement of the group’s gait speed (10MWT) from MFCL K2 to MFCL K3 as a result of the longitudinal intervention. The lines indicating K2 and K3 in the plot are established benchmark from literature

In comparison to baseline gait speed, 66% of the participants improved their gait speed above K3 gait speed (i.e. previously published gait speeds averages of individuals at K3 level), when using the MPK C-Leg + 1M10. While, only 33% of the participants using the NMPK and 1M10 feet improved their gait speed above K3 level gait speed (Fig. 2).

#### Balance

**Fig. 3:**
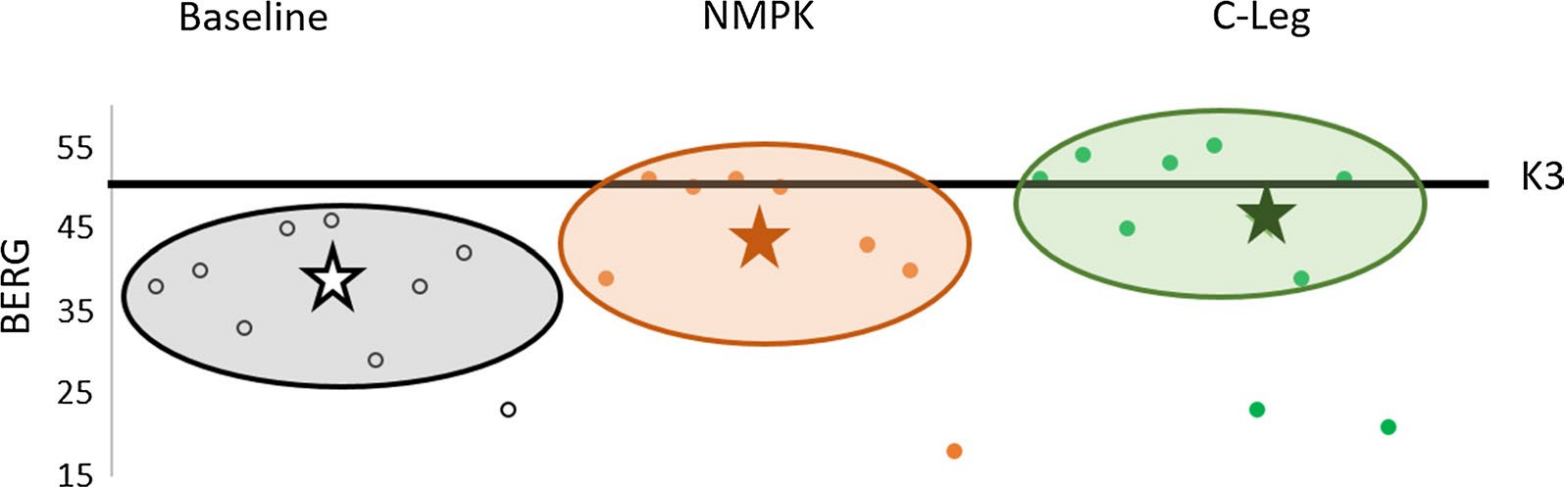
Performance improvement of the group’s Berg balance scores from MFCL K2 to MFCL K3 as a result of the longitudinal intervention

Participant balance scores improved to values within the range of scores achieved by individuals with K3 functional level (BERG ≥ 50.5/56) when using the MPK C-Leg combination (baseline = 37 (8), MPK C-Leg = 44 (13), NMPK = 39 (15)). However, there was no change noted with the NMPK + 1M10 intervention (Fig. 3).

#### AMPPro

A statistically significant effect was observed for AMPPro [F(2,26) = 6.69, P = 0.008, power = 0.78]. Follow-up posthoc tests showed statistically significant improvement for AMPPro scores from baseline for both the interventions (MPK C-Leg and NMPK) ([t(16) = 3.62, P = 0.018]_Baseline vs. MPK C-Leg_, [t(16) = 2.99, P = 0.03]_Baseline vs. NMPK_: Baseline = 31 (7), MPK C-Leg = 36 (5), NMPK = 35(6))_AMPPro_. Compared to baseline, both interventions lead to clinically meaningful improvement in group mean AMPPro scores (MDC_90_ = 3.4 [29]). However, this improvement was not high enough to match K3 level for both interventions (K2 level: 34.7 ± 6.5; K3 level: 40.5 ± 3.9, [30]).

### Participant-reported outcome measures

Participants-reported scores showed statistically significant improvements [F(2,26) = 9.02, P = 0.003, power = 0.90; baseline = 60.63 (18.75), MPK C-Leg = 81.92 (18.74), NMPK = 59.15 (19.31)]_PEQ-A_ and [F(2,26) = 8.52, P = 0.004, power = 0.88; baseline = 7.78 (1.14), MPK C-Leg = 9.33 (0.69), NMPK = 8.51 (1.03)]_MFES_.

#### PEQ-A

Follow-up post-hoc tests between baseline and intervention revealed statistically significant higher scores for PEQ-A while using the MPK C-Leg compared to baseline (t(16) = 3.62, P = 0.008, baseline = 60.63 (18.75), MPK C-Leg = 81.92 (18.74))_PEQ-A._ This implies that participants perceived MPK C-Leg + 1M10 provided a greater ability to walk on different terrain and surfaces (level ground, stairs, closed spaces, slippery surfaces, steep inclines, and sidewalks) in comparison to their NMPK. In comparison to baseline scores, 78% of the participants reported higher PEQ-A scores while using MPK. These improved scores matched previously published literature of higher K3 MFCL performance level when using the MPK C-Leg. No improvement from baseline scores was observed while using NMPK + 1M10 for PEQ-A (baseline = 60.63 (18.75), NMPK = 59.15 (19.31)).

#### MFES

**Fig. 4:**
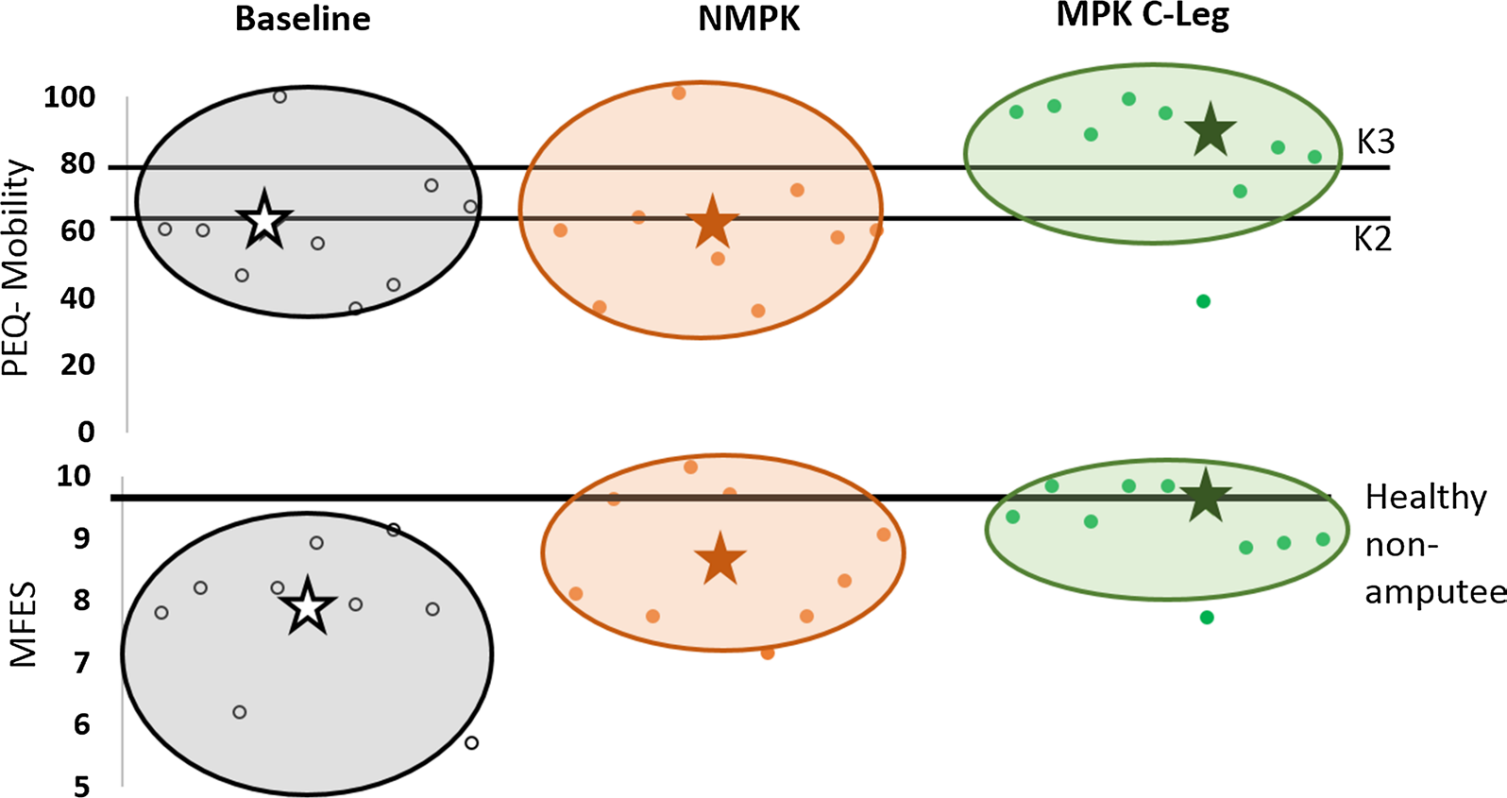
Performance improvement of the group’s safety (MFES) and self-reported mobility scores from MFCL K2 to MFCL K3 as a result of the longitudinal intervention

Similarly, post-hoc tests showed that participants reported significantly improved MFES score when using an MPK C-Leg + 1M10 in comparison with their baseline MFES score (t(16) = 4.14, P = 0.003, baseline = 7.78 (1.14), MPK C-Leg = 9.33 (0.69), NMPK = 8.51 (1.03)). There was no statistically significant improvement in MFES scores from the baseline condition when using the NMPK (P > 0.05). This implies that the participants perceived reduced fall risk while using the MPK C-Leg and foot combination. Most participants reported MFES scores as high as healthy individuals (healthy non-amputees) when using the MPK C-Leg + 1M10, implying fall risk and fear of falling reduced significantly (Fig. 4).

## Discussion

This prospective randomized crossover longitudinal clinical trial investigated the central hypothesis that individuals with transfemoral amputation from dysvascular conditions, designated at K2 MFCL level (and currently using an NMPK leg + foot combination) can elicit clinically relevant improvements to gait performance and safety from using an MPK C-Leg + 1M10 foot.

Unique to our clinical trial was the study design undertaken. Our protocol addressed some of the research gaps identified by a policy consensus investigation [24]. 50% of the participants were elderly with transfemoral amputation from dysvascular conditions designated at the K2 level (limited community ambulators). The study design quality matched an A grade based on Hofstad rating: (Additional file 1: Table S1) for prosthesis related research. Further, the protocol adopted for device training for acclimation and evaluation periods were standardized between both interventions (Block_MPK_ and Block_NMPK_) and we also controlled for co-interventions (used the same foot type 1M10 for both interventions—Block_MPK_ and Block_NMPK_). We collected an extensive list of outcome measures that includes clinical performance-based measures, participant-reported outcome measures, and used a priori established ranges of values from prosthetic literature to facilitate clinical inference.

This longitudinal clinical trial evidence shows that using an MPK C-Leg + appropriate foot, coupled with device acclimation, improved performance in many participants from a K2 MFCL to match a K3 MFCL. Specifically, the majority of the K2 participants in our group could perform at K3 MFCL in four aspects of performance, namely, gait speed (m/s—10MWT), balance metrics (BBS), improved mobility scores (PEQ-MS), and reduced fall risk (MFES). Compared to performance at baseline condition, both interventions led to a similar degree of improvement in AMPPro group mean scores.

These findings are of significant importance in this group of generally deconditioned older adults where an increase in walking speed, participant-reported outcome scores, and reduced fall risk can be associated with improved quality of life [18, 19, 32–34]. Furthermore, our results showing the benefits of using an MPK leg are also consistent with past literature on individuals with transfemoral amputations [18].

Previous research has shown strong evidence that using an MPK has led to improved performance in measures of balance and gait in individuals with transfemoral amputations who used an NMPK as their predicate everyday device [14, 18, 19, 35]. However, most of this previous literature had a small representation of older amputees due to vascular disease at K2 level. A notable exception is a large clinical trial from 2018 [19], which showed the benefits of using MPK in a group of patients similar to our study. However, the clinical trial design adopted by Kaufmann et al. 2018 was not longitudinal in nature [19]. All this pioneering evidence [14, 18, 27, 35–37] from experts in the prosthetic community has been valuable for us to benchmark our findings. We hope our study will further strengthen the evidence base and potentially impact policy-level decisions to improve healthcare access in K2-level transfemoral amputees using an NMPK.

From a technological capability standpoint, potential reasons for these observed improvements can be attributed to the MPK (C-Leg) + 1M10 combination. The C-Leg controller houses sensors at the knee to provide feedback on dynamic ambulation conditions to the microprocessor, thus providing a more stable and modulated gait (variable cadence, etc.) to suit individual user-specific needs. Further, the C-Leg also has advanced functionality like stumble recovery which reduces fall risk in comparison to the passive mechanical knees investigated. An increase in gait speed combined with the versatile functionality of the C-Leg to facilitate navigation in different terrains could have contributed to the improved overall satisfaction for function, perhaps allowing more confident mobility. Furthermore, the 1M10 foot was complementary to the C-Leg providing the stability and safety required by K2 users to confidently use an advanced prosthesis.

The studied group did not show statistically significant improvements for some of the clinical performance outcomes including 6MWT, FSST, and TUG. This could be attributed to the variability in the predicate knee types (NMPK) (Table 1) or the lower performance ability of the participants themselves in terms of endurance and balance. It is also possible that the study was underpowered to show statistically significant differences for these clinical outcome measures as the study was originally powered based on large data metrics (GPS variables).

## Limitations

It was observed that there may be a benefit of only switching foot type to 1M10 to improve functional outcomes (i.e. NMPK intervention). 33% of the group improved in gait speed to the performance level of K3 from the NMPK + 1M10 intervention (after receiving training for acclimation). However, our small sample size and study design goal determined a priori were not conducive to observe this effect. We hope to revisit this in future investigations. We standardized the foot type to 1M10 for everyone during the interventions (Block_MPK_ and Block_NMPK_) to reduce the effect of variable predicate foot type on the overall study outcomes, and despite the variability in predicate NMPK, this group elicited significant benefit from using a C-Leg MPK. Other study designs could also have been considered, including comparing the MPK + 1M10 group vs. NMPK + 1M10 group or MPK + 1M10 vs. MPK + predicate feet directly, instead of comparing with the baseline devices. An acclimation period of 14 visits was allowed over 3 months for training prior to home trials. Even though the authors believe this only helped for learning how to safely use of the prostheses at home, there is a potential for additional benefits which could have influenced the study results. The mean age of study participants is 63 ± 9 years, which is relatively young when compared to the typical age range (70–75 years) of transfemoral amputation due to vascular complications in the United States [38]. Further, typically this population has a higher incidence of comorbidities such as diabetes, cardiovascular/heart disease, and peripheral artery disease [39]. We did not document any information regarding the use of assistive devices outside the clinical facility (i.e. home). Advanced wearable sensor-based activity recognition can be beneficial in future studies to capture this information. A larger multi-site clinical trial, controlling for predicate knee types, homogenous groups in terms of demographics, and secondary medical conditions is warranted. Larger sample size is recommended to replicate and expand these findings for generalization to the study population. Future studies should include specific outcomes to track performance on uneven terrain, stairs, community ADLs, and aspects of health economics.

## Conclusions

This longitudinal clinical trial investigated the benefit of providing an MPK C-Leg + appropriate foot in individuals with transfemoral amputation from dysvascular or diabetic conditions at MFCL K2 level who are currently using a predicate NMPK + foot combination. Statistically significant and clinically meaningful improvements were observed in gait performance, safety, and self-reported measures (PEQ-A) when using the MPK C-Leg + 1M10 foot combination in comparison to their baseline condition (i.e. predicate NMPK + foot).

### Supplementary Information

**Additional file 1: Table S1.** Hofstad study statistical design quality rating.

**Additional file 2: Figure S1.** Ottobock® C-Leg and 1M 10 foot.

**Additional file 3: Figure S2. **Sample size and power estimation.

## Data Availability

De-identified data are available from the authors upon reasonable request.

## Appendix

### Device

#### C-Leg

The C-Leg features programmable modes for special movement patterns (e.g. cycling, golf, etc.) that require knee resistances outside of typical gait swing and stance control. Other key features include a safety mode allowing for restricted operation in case of walking with a battery that has been depleted. It can provide stumble recovery by providing high resistance following a misstep to prevent knee buckling in a trip/fall scenario. Thus, in comparison to a passive mechanical knee, the C-Leg is versatile in facilitating a wide range of functional activities including walking on ramps, stairs, and uneven terrain [32, 33] (Additional file 2: Figure S1).

### Sample size estimation

This clinical trial was the first to do a long-term home monitoring GPS based study design in prosthetics literature. So there was no information from prosthetic literature for sample size estimation using the GPS metric. Therefore we used GPS reference outcomes for sample size estimation based on discussion and reference values from other literature [*Physiotherapy Canada; 65(3);279–288; *https://doi.org/10.3138/ptc.2012-36*, ORIGINAL ARTICLE Community activity and participation are reduced in transtibial amputee fallers: a wearable technology study Brenton Hordacre, Christopher Barr, Maria Crotty*], which is distance covered outdoors of 580 m.

For the discussion regarding transition from K2 to K3, we used the following reference from literature [29] to get the mean and standard deviation differences between K2 and K3 amputee population for a 6MWT (112 m and ~ 96 m respectively). Based on these values the sample size and power calculation were calculated for matched samples.

The sample size (n = 9) was estimated to achieve a power of 80% and a level of significance of 5% (two sided) for detecting a mean of the differences of 112 between pairs, assuming the standard deviation of the differences to be 96. Therefore our GPS study sample size was n = 10. The sample size estimation plots for a range of paired differences in SD is shown (Additional file 3: Figure S2).

### Clinical trial quality assessment

The Hofstad’s checklist comprises 13 criteria to assess methodological quality that is all scored either: no = 0, yes = 1, or not applicable. The scoring rules used for the individual criterion can be found in Hofstad et al. [27]. Based on the Hofstad score, a clinical trial in prosthetics is rated as, A grade (high quality), B grade (moderate quality) and C grade (low quality).

The clinical trial process was reviewed and rated based on the [26] rating supplemental table which shows the rated scores for each of the 13 items on the checklist (A1 through C13). Based on the assessed scores, this clinical trial quality was rated as an A grade (high quality). Two of the authors performed quality assessments independently. On completion of the individual assessment, the scores were formally discussed and reviewed with a certified Prosthetists and Physical Therapist. Any disagreement in scores was discussed and corrected with the consensus of the group following this discussion. Additional file 1: Table S1 shows the corrected scores.

*A grade (high quality)* If the clinical trial reaches a score of 11 points in total, with at least 6 points in the participant selection (A) and intervention (B) criteria with valid scores in blinding (B7) and accommodation (B8). *B grade (moderate quality)* If the clinical trial reaches a score of a minimum of 6 points in total, with at least 6 points in the participant selection (A) and intervention (B) criteria with a valid score in accommodation (B8). *C grade (low quality)* If the clinical trial reaches a score of a minimum of 6 points in total, with at least 6 points in the participant selection (A) and intervention (B) criteria with invalid scores in blinding (B7) and accommodation (B8).

The quality of the clinical trial design was scored for the following criteria.

### Criteria for methodological quality

#### Selection of participants

1. Adequate description of inclusion and exclusion criteria (with a minimum of three from the following descriptors: age, amputation level, etiology, level of activity, time since amputation, residual limb condition, comorbidities, and sex)?
2. Homogeneity of the study groups (at least for age, etiology, amputation, and mobility levels)?
3. Prognostic comparability of the study sample (e.g., for etiology and amputation level, age, sex, condition of the residual limb, comorbidities, etc.; prognostic comparability is given by definition in within-subject studies with every participant acting as their control)?
4. Randomization (randomized order of intervention: 1 point, randomization of participants to intervention and control groups [RCT]: 2 points)?

#### Intervention

B5. Description of experimental intervention (study repeatability)?
B6. Control of co-interventions?
B7. Blinding of participants and/or assessors?
B8. Timing of measurement (adequate adaptation)?
B9. Appropriateness of outcome measures to answer the research question of the study?

#### Statistical validity

C10. Attrition rate under 20%?
C11. Adequate sample size (sample size calculation and power analysis)?
C12. Intention-to-treat analysis?
C13. Data presentation (point estimates and measures of variability)?
