# Supplementary material for: Using a microprocessor knee (C-Leg) with appropriate foot transitioned individuals with dysvascular transfemoral amputations to higher performance levels: a longitudinal randomized clinical trial
Source: J Neuroeng Rehabil. 2021 May 25;18:88. doi: 10.1186/s12984-021-00879-3 (PMC8146219; doi:10.1186/s12984-021-00879-3)
Supplement: Supplementary file 1 — Additional file 1: Table S1. Hofstad study statistical design quality rating. [file 12984_2021_879_MOESM1_ESM.docx]

| **Hofstad Criteria** | **Hofstad criteria evaluated** | **Section to refer** | **Hofstad Rating** | **Total score** | **Hofstad Criteria total** | **Overall grade for the quality of clinical trial based on Hofstad rating** |
| --- | --- | --- | --- | --- | --- | --- |
| Selection of Patients | A1 | Methods,  Demographics table | 1 | A_total_ =4 | (A+B)=9     4(A+B+C)=11 | (A+B)>6; (A+B+C)<11 and B8=1. |
|  | A2 |  | 1 |  |  |  |
|  | A3 |  | 1 |  |  | **Rating: A Grade** (High Quality) |
|  | A4 |  | 1 |  |  |  |
| Intervention | B5 | Methods-  Clinical trial design | 1 | B_total_=5 |  |  |
|  | B6 |  | 1 |  |  |  |
|  | B7 |  | 1 |  |  |  |
|  | B8 |  | 1 |  |  |  |
|  | B9 |  | 1 |  |  |  |
| Statistical validity | C10 | Methods-  Study data analysis | 1 | C_total_=2 |  |  |
|  | C11 |  | 0 |  |  |  |
|  | C12 |  | 0 |  |  |  |
|  | C13 |  | 1 |  |  |  |
|  |  |  |  |  |  |  |
