# Supplementary figures and images for: Using a microprocessor knee (C-Leg) with appropriate foot transitioned individuals with dysvascular transfemoral amputations to higher performance levels: a longitudinal randomized clinical trial
Source: J Neuroeng Rehabil. 2021 May 25;18:88. doi: 10.1186/s12984-021-00879-3 (PMC8146219; doi:10.1186/s12984-021-00879-3)

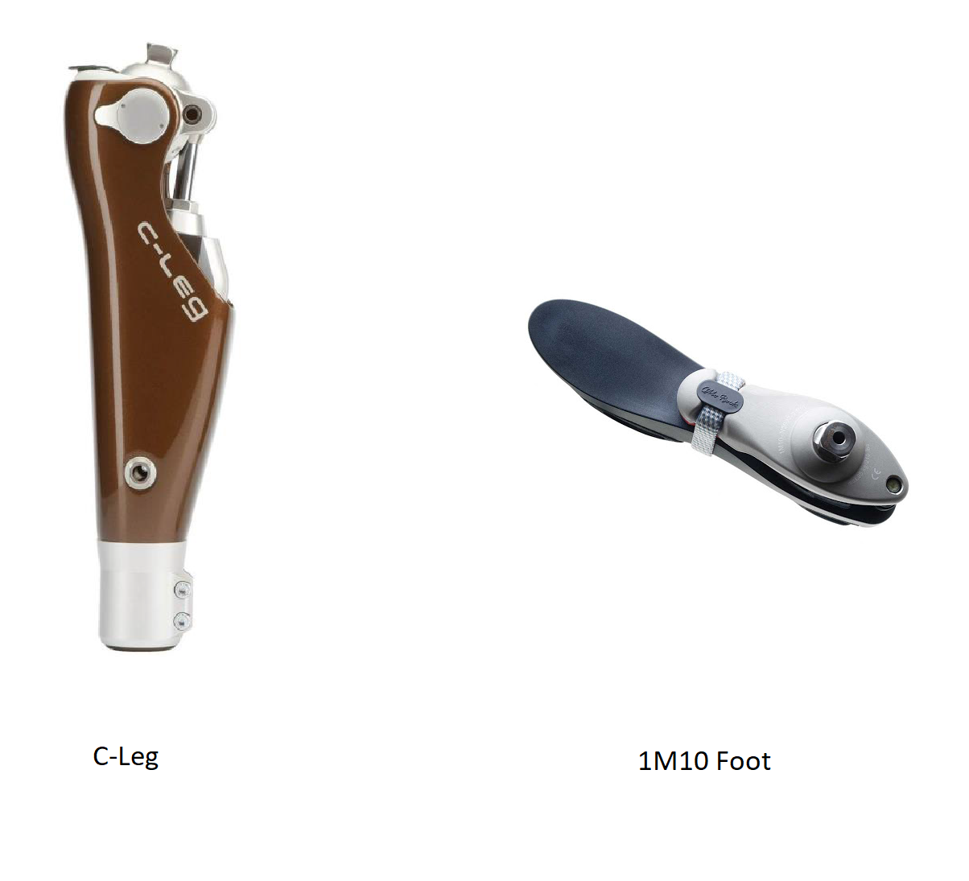

Supplement: Supplementary file 2 — Additional file 2: Figure S1. Ottobock® C-Leg and 1M 10 foot. [file 12984_2021_879_MOESM2_ESM.tif]
